# Microbial Degradation of Nicotinamide by a Strain *Alcaligenes* sp. P156

**DOI:** 10.1038/s41598-019-40199-0

**Published:** 2019-03-06

**Authors:** Chunhui Hu, Shuxue Zhao, Kuiran Li, Hao Yu

**Affiliations:** 1 Shandong Provincial Key Laboratory of Applied Mycology, College of Life Sciences, Qingdao Agricultural University, Qingdao, China; 2 College of Marine Life Science, Ocean University of China, Qingdao, China

## Abstract

A novel *Alcaligenes* sp. strain P156, which can utilize nicotinamide as its sole source of carbon, nitrogen and energy, was enriched and isolated from soil in a solid waste treatment plant. Aerobic growth and degradation with nicotinamide were characterized. Seven nicotinamide degradation-related genes were obtained by sequence alignment from the genome sequence of strain P156. Four genes, designated *naaA*, *naaD*, *naaE* and *naaF*, were cloned and heterologously expressed. Nicotinamide degradation is initiated by deamination to form nicotinic acid catalyzed by the nicotinamidase NaaA, which shares highest amino acid sequence identity (27.2%) with nicotinamidase from *Arabidopsis thaliana*. Nicotinic acid is converted to 6-hydroxynicotinic acid, which is further oxidized to 2,5-dihydroxypyridine (2,5-DHP). 2,5-DHP is then transformed to a ring-cleavage product, *N*-formylmaleamic acid, by an Fe^2+^ dependent dioxygenase NaaD. *N*-formylmaleamic acid is transformed to fumaric acid through maleamic acid and maleic acid by NaaE and NaaF, respectively. To our knowledge, this is the first report of the complete microbial degradation of nicotinamide in bacteria. Nicotinamide is considered as a model compound for the study of microbial degradation of pyridinic compounds, and the nicotinamide degrading related genes in strain P156 were distributed differently from the reported similar gene clusters. Therefore, this study contribute to the knowledge on the degradation of pyridinic compounds.

## Introduction

Pyridine and its derivatives compose one of the largest classes of known organic chemicals. The nitrogen atom makes pyridine ring more electrophilic, which gives pyridinic compounds outstanding biological activities. Therefore, pyridinic compounds are widely used in chemical, agricultural, pharmaceutical, and food industries. In nature, pyridine ring can be found in coenzymes, plant alkaloids, and the secondary products generated by microorganisms^1–3^. Just like its homocyclic analogs, pyridinic compounds are considered persistent pollutant and most of them are harmful to human and other organisms and classified as priority pollutants by United States Environmental Protection Agency^4–6^. Pyridine and its derivatives are easy to spread in environment due to the water solubility property^7^. Therefore it is necessary to remove pyridinic compounds from the environment. Pyridinic compounds can be removed by physical^8,9^, chemical or biological methods. Microbial biodegradation could remove these compounds efficiently without secondary pollution^4,5,7^, therefore, microorganisms play significant roles in degradation of pyridine derivatives^7,10^.

Nicotinamide, also known as a form of vitamin B3, is a part of the coenzyme nicotinamide adenine dinucleotide (NADH/NAD^+^) and is crucial to life. Nicotinamide is the most distributed and commonly used pyridinic compounds found in food, dietary supplement, cosmetics and medication^11^. Although nicotinamide was widespread in the environment, information of microbial degradation of nicotinamide is quite limited. The study of microbial transformation of nicotinamide mainly focus on the first deamination step^12,13^. In the deamination step, nicotinamide was transformed to nicotinic acid by nicotinamidase releasing NH_4_^+ ^^13^. This reaction is not only involved in NAD^+^ biosynthesis, but in many other important physiological processes. The product, nicotinic acid, does not seem to influence these physiological processes, therefore, the fate of nicotinic acid in these organisms were not reported^11,14^. Nicotinamide is ubiquitous in all living organisms, thus, degradation of nicotinamide could be considered as model systems for the degradation of pyridinic compounds. Microbial degradation of nicotinamide will help us to understand the degradation processes of other pyridinic pollutants, such as picloram and diquat^7,15^.

In this study, we have isolated a nicotinamide degrading bacterium, *Alcaligenes* sp. P156, from the soil in solid waste treatment plant. The genome sequence of strain P156 was determined, and a DNA fragment was predicted to be involved in nicotinamide degradation. The functions of four genes were characterized in detail. The objective of this work is to demonstrate the nicotinamide catabolism in bacteria.

## Results

### Isolation and Identification of *Alcaligenes* sp. strain P156

The bacterial strains were isolated from contaminated soil in a solid waste treatment plant. The strain, which could use picolinic acid (PIA) and nicotinamide as the sole source of carbon, nitrogen, and energy, was obtained by plate streaking with enrichment cultivation and designated as P156 (Table 1). The 16S rRNA gene of strain P156 exhibits high sequence similarity with those of *Alcaligenes* strains, having the highest similarity of ~97% with *Alcaligenes faecalis* subsp. *parafaecalis*. Strain P156 was identified as a Gram-negative, aerobic strain and classified as *Alcaligenes* sp. Isolated P156 strain has been deposited in the China Center For Type Culture Collection, Wuhan under the accession numbers CCTCC AB 2016309.

**Table 1:** Growth and degradation of strain P156 with different substrates.

Substrates	Growth	Degradable
2-hydroxypyridine	ND^a^	ND
3-hydroxypyridine	ND	ND
4-hydroxypyridine	ND	ND
picolinic acid	+	+
nicotinic acid	+	+
isonicotinic acid	ND	ND
nicotinamide	+	+
6-hydroxynicotinic acid	+	+
2,3-dihydroxypyridine	ND	ND
2,5-dihydroxypyridine	ND	+
2,6-dihydroxypyridine	ND	ND

All the experiments were performed in MSM with 1 mg/mL corresponding substrate.

^a^ND, not detectable.

### Co-degradation of PIA and nicotinamide by strain P156

**Figure 1:**
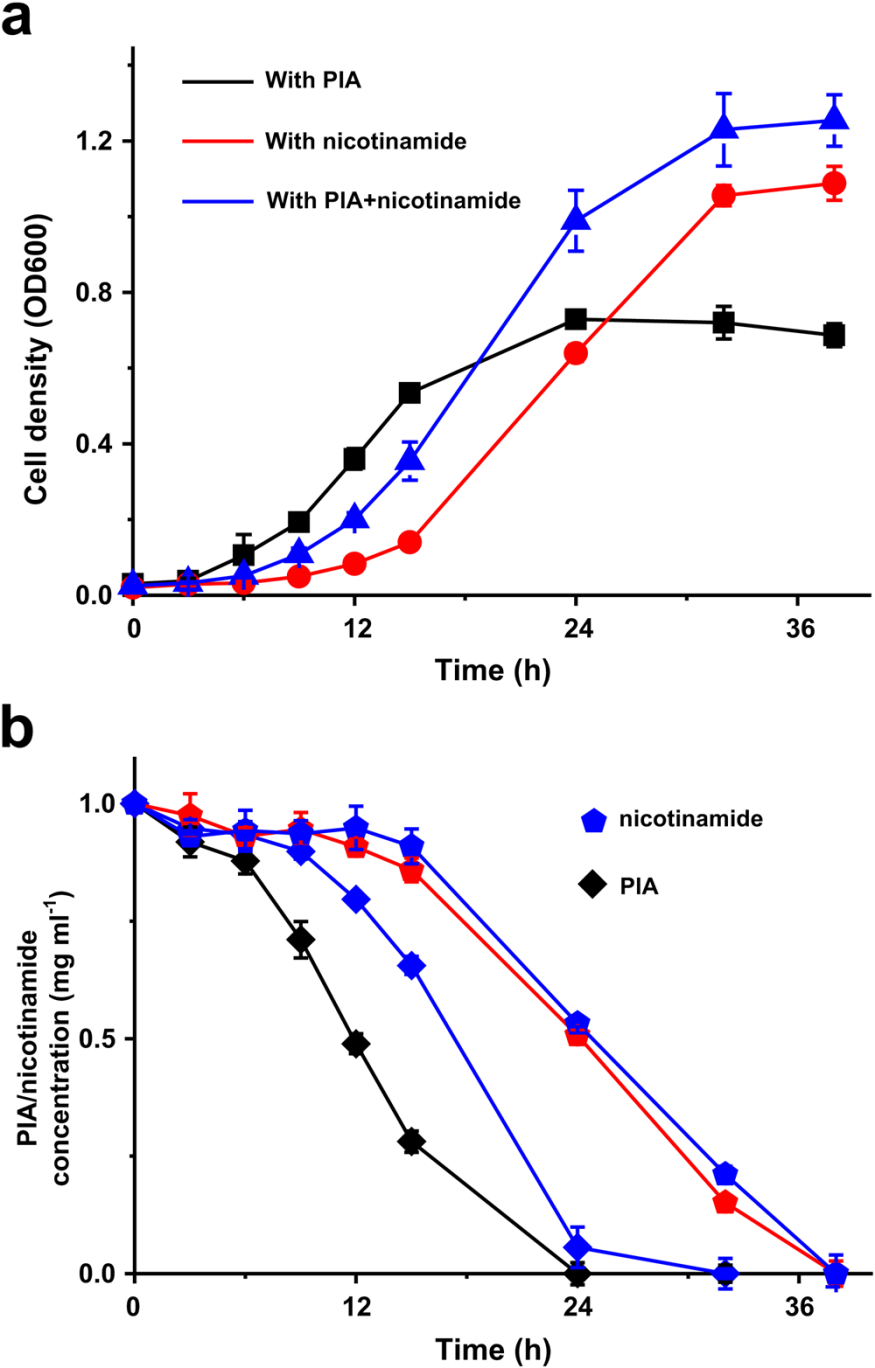
Degradation of PIA, nicotinamide and the growth of strain P156. (**a**) The growth of strain P156 in MSM medium supplemented with 1 mg/mL PIA (black line), 1 mg/mL nicotinamide (red line) and a mixture of 1 mg/mL PIA and 1 mg/mL nicotinamide (blue line). (**b**) PIA (diamond) and nicotinamide (pentagon) degradation in different media by strain P156. Each value is the mean of results from three parallel replicates ± the standard deviation (SD).

Strain P156 was cultivated with PIA, nicotinamide and a mixture of PIA and nicotinamide. As we can see from Fig. 1, the growth of strain P156 reached a maximum with the mixture substrates compared with the single substrate (Fig. 1a). Strain P156 has the shortest lag phase when grow with PIA; however, the final biomass of strain P156 with PIA is the smallest. PIA and nicotinamide degraded simultaneously in the culture with mixed substrates. PIA biodegradation seemed to be slightly slowed down in the presence of nicotinamide; however, the degradation rate of nicotinamide was basically the same in the presence or absence of PIA (Fig. 1b).

**Figure 2:**
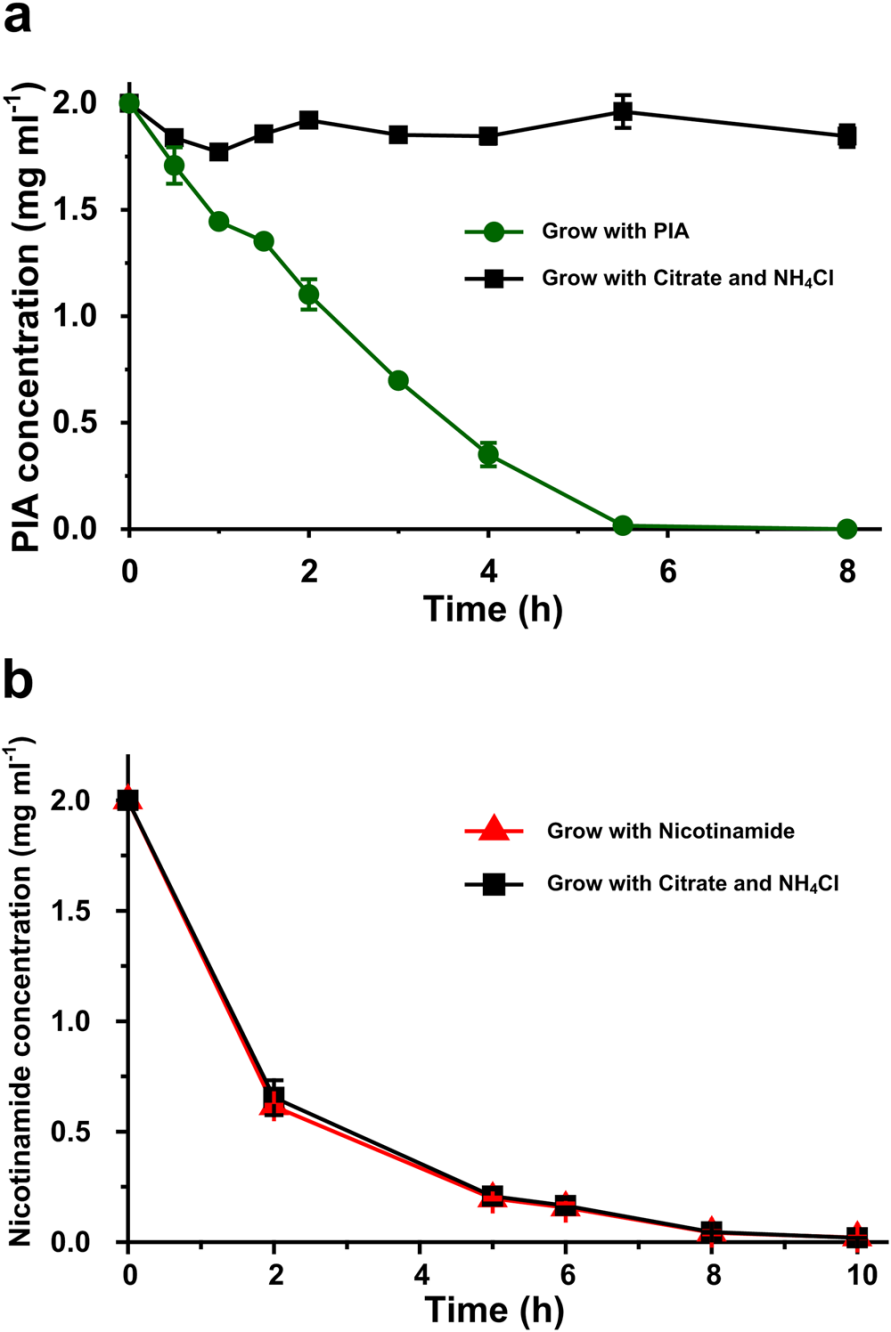
PIA and nicotinamide degradation by resting cells of strain P156. (**a**) PIA degradation by resting cells of strain P156 prepared from MSM with PIA (green line) and citrate/NH_4_Cl media (black line). (**b**) Nicotinamide degradation by resting cells of strain P156 prepared from MSM with Nicotinamide (red line) and citrate/NH_4_Cl media (black line). Each value is the mean of results from three parallel replicates ± the SD.

PIA and nicotinamide degradation reactions were performed by resting cells of strain P156 prepared from MSM + PIA/nicotinamide or MSM + NH_4_Cl + sodium citrate, respectively. PIA was completely transformed within 6 hours by PIA-induced resting cells of strain P156; however, PIA concentration in reaction with non-PIA induced cells was basically unchanged (Fig. 2a). Unlike PIA, the nicotinamide was completely transformed within 8 hours by resting cells of strain cultivated with nicotinamide or NH_4_Cl + sodium citrate (Fig. 2b). Besides, no nicotinic acid was accumulated in the samples from resting cell reactions. The results indicated that the expression of enzyme responsible for PIA conversion was induced by PIA. However, the enzyme responsible for nicotinamide conversion was expressed constitutively.

### Growth kinetics with nicotinamide

**Figure 3:**
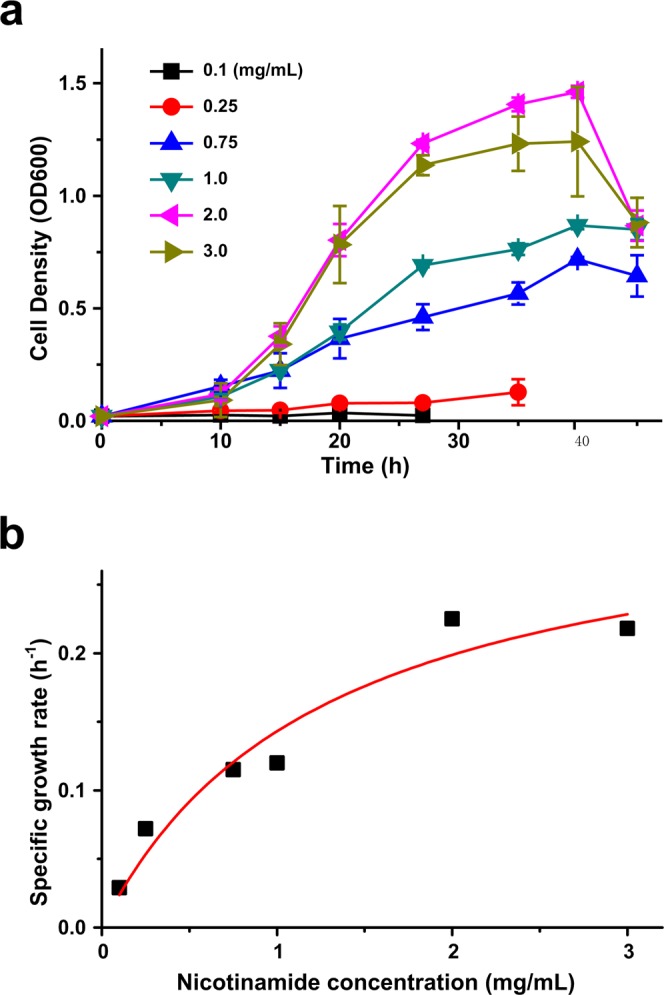
Growth and specific growth rate of *Alcaligenes* sp. P156. (**a**) Growth of strain P156 with different concentrations of nicotinamide at 30 °C pH 7.0. (**b**) Monod’s growth model fitted to results of batch growth experimental data to determine the growth kenetics parameters of strain P156. Each value is the mean of results from three parallel replicates the SD.

In the batch experiment, the growth of strain P156 increased with the increase of nicotinamide concentration. The lag phase was basically unchanged with different concentration of nicotinamide (Fig. 3a). The specific growth rate (*μ*) and initial concentration of nicotinamide have been plotted in Fig. 3b. Kinetic constants were estimated by specific growth rate fitted to Monod’s model and Haldane inhibitory growth model. It has been found that Monod’s model can represent the date of entire region (Fig. 3b), which indicates that nicotinamide have no inhibitory effect on growth of strain P156 in this range. The maximum specific growth rate (*μ*_max_) and half saturation (*K*_s_) are 0.32 ± 0.06 h^−1^ and 1.27 ± 0.53 mg/mL, respectively.

### Nicotinamide degradation by *Alcaligenes* sp. P156

**Figure 4:**
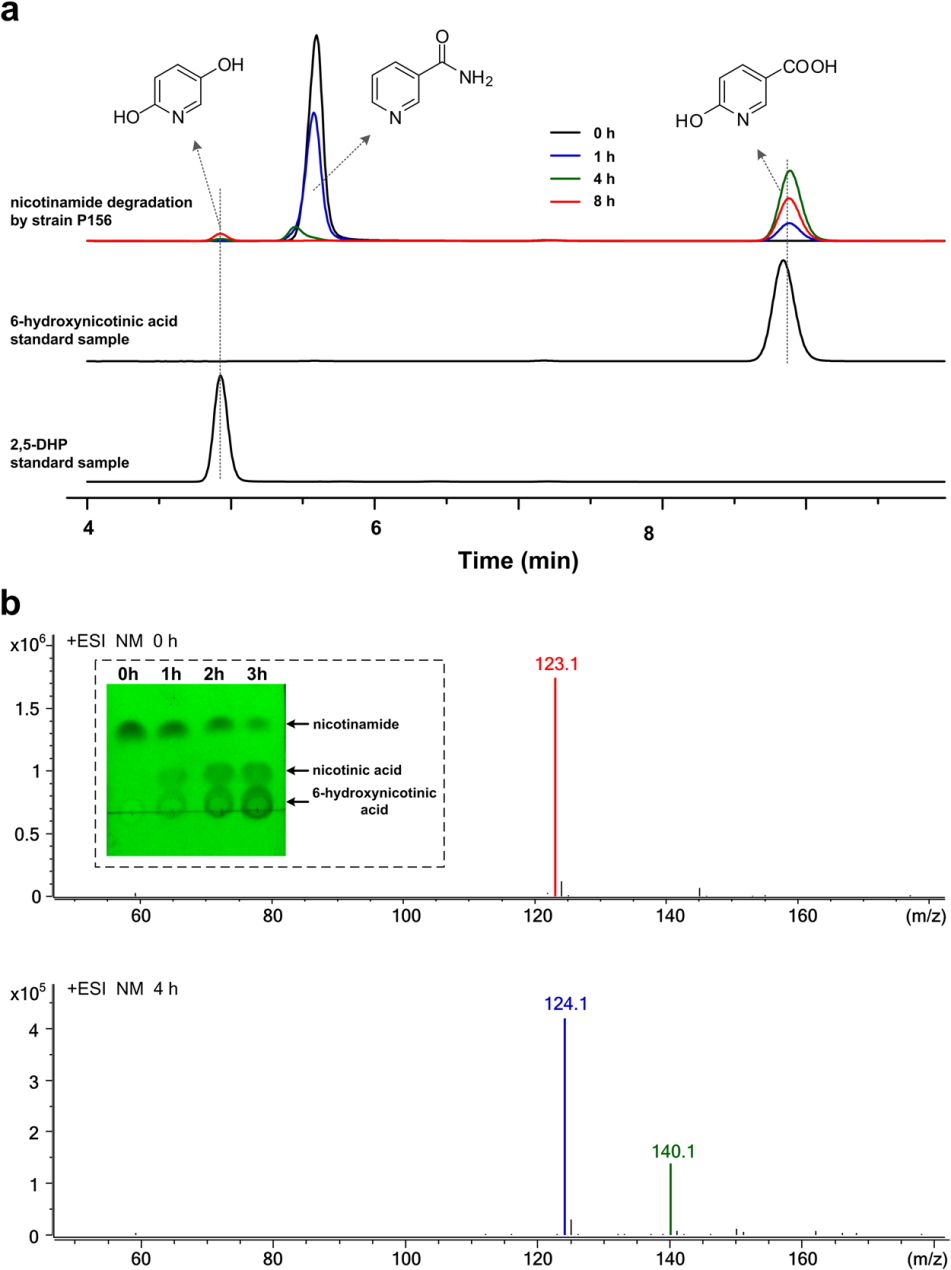
HPLC and ESI-MS analysis of nicotinamide degradation by *Alcaligenes* sp. P156. (**a**) The reaction samples at 0 h (black line), 1 h (blue line), 4 h (green line) and 8 h (red line) for the degradation of nicotinamide by resting cells of *Alcaligenes* sp. P156. (**b**) LC-MS analysis of the reaction samples at 0 h and 4 h. The structure of the compounds have been indicated in the figure. The inset is the photograph of TLC plate (visualized in UV light) with reaction samples at 0 h, 1 h, 2 h and 3 h. The compounds, represented by the spots, were indicated on the right of TLC plate (full length spots can be observed in Supplementary Information).

Strain P156 could use nicotinamide as the sole source of carbon, nitrogen and energy. To confirm the degradation pathway of nicotinamide in strain P156, time course biotransformation of nicotinamide by resting cells of *Alcaligenes* sp. P156 was analyzed by HPLC and LC-MS. When the signal representing nicotinamide decreased, two new peaks emerged in the HPLC signal. The retention time and spectra of the new products resemble those of 6-hydroxynicotinic acid and 2,5-dihydroxypyridine (2,5-DHP), respectively (Figs 4a, S1 and S2). When use 6-hydroxynicotinic acid as the substrate, 2,5-DHP was detected by HPLC (data not shown). Chromatograms of resting cell reaction samples on thin-layer chromatography (TLC) are presented in Fig. 4b. It can be seen that the spots representing nicotinamide shallowed, and the spots representing nicotinic acid and 6-hydroxynicotinic acid emerged and deepened gradually. In LC-MS results, signal representing the molecular mass of nicotinamide ([M-H]^−^, *m/z* 123.1) disappeared at 4 h, the new signals representing nicotinic acid ([M-H]^−^, *m/z* 124.1) and 6-hydroxynicotinic acid ([M-H]^−^, *m/z* 140.1) were observed (Fig. 4b).

**Figure 5:**
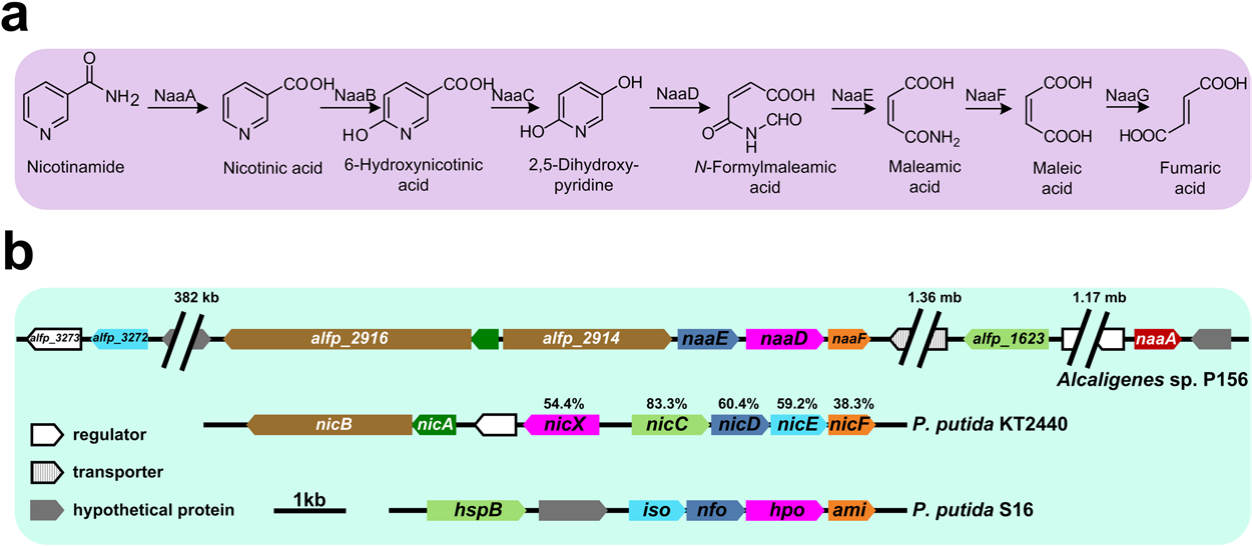
Proposed pathway of nicotinamide degradation in *Alcaligenes* sp. P156. (**a**) Proposed nicotinamide degradation pathway in *Alcaligenes* sp. strain P156. (**b**) Physical map of the DNA fragment containing genes involved in nicotinamide degradation in strain P156. The arrows indicates the location, direction and size of the transcription of the ORFs.

Based on HPLC and LC-MS results, it can be concluded that nicotinamide was initially transformed into nicotinic acid, then the nicotinic acid was further transformed through 6-hydroxynicotinic acid and 2,5-DHP (Fig. 5a). To accelerate the study of molecular mechanism, the genome of strain P156 was sequenced. Several genes were found in the genome of strain P156 that encode proteins showing amino acids sequence identity with reported nicotinic acid or nicotine degradation proteins from *P. putida* KT2440 or *P. putida* S16^11,16,17^. ALFP_1623 shows 83.3% sequence identity with 6-hydroxynicotinic acid (NicC) from *P. putida* KT2440, and is predicted to catalyze the hydroxylation of 6-hydroxynicotinate. The deduced amino acid sequence of the gene *naaD* shares 54.4% sequence identity with NicX from strain KT2440, indicating that the gene may be responsible for the conversion of 2,5-DHP to *N*-formylmaleamic acid. Proteins from NaaE to NaaG were supposed to catalyze the conversion of 2,5-DHP to fumaric acid (Fig. 5a,b).

### The *naaA* gene encodes the nicotinamidase

**Figure 6:**
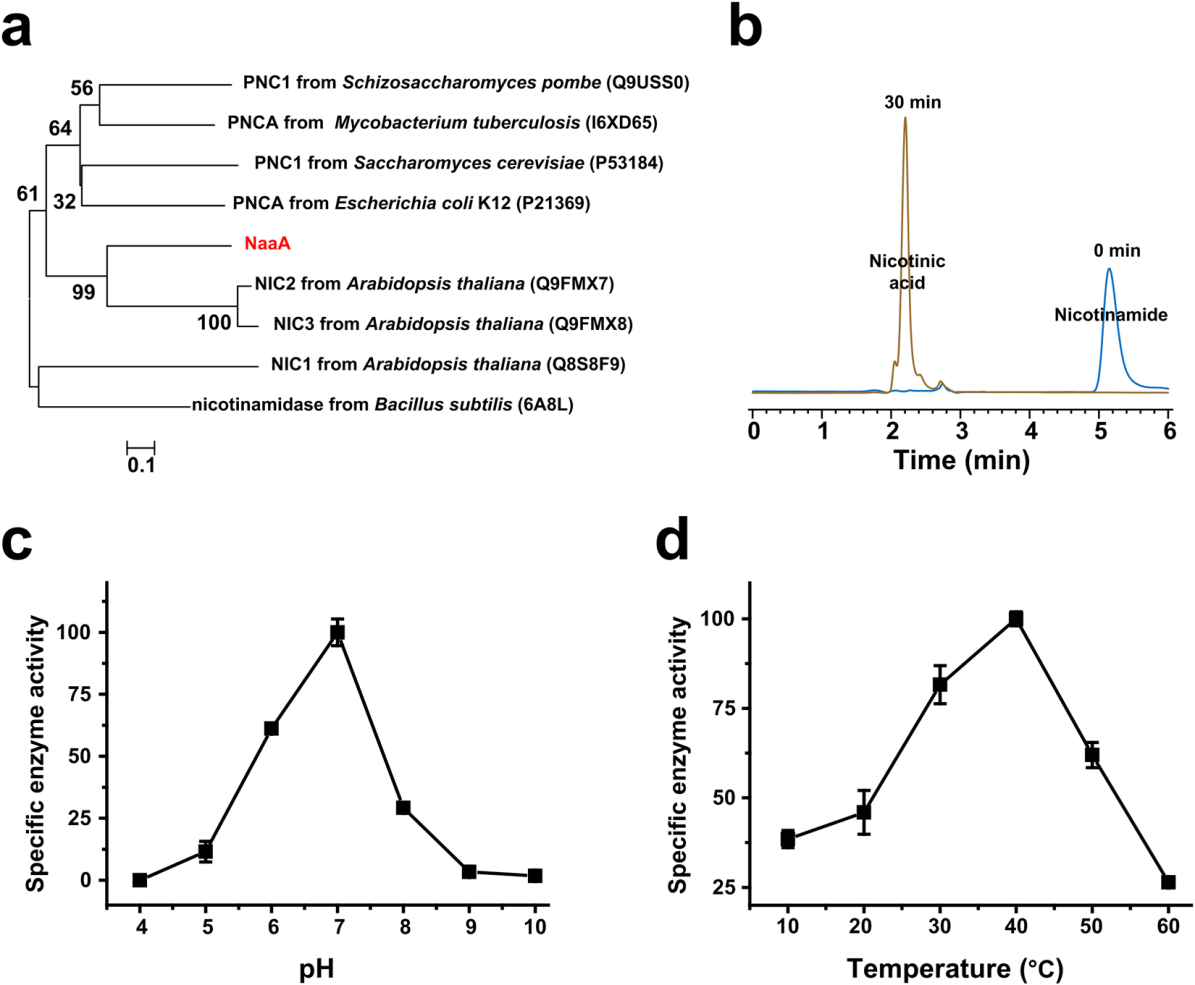
Characterization of NaaA. (**a**) Phylogenetic analysis of NaaA and related nicotinamidases. The GenBank accession number for each protein is shown in parentheses. (**b**) HPLC analysis of the reaction of NaaA at 0 min and 30 min. (**c**) pH-dependent enzyme activity of NaaA. (**d**) Temperature dependent enzyme activity of NaaA. Each value is the mean from three parallel replicates ± SD.

The first enzymatic step in nicotinamide degradation is nicotinamide deamination. This step was catalyzed by nicotinamidase, which has not been studied in pyridinic compound degradation strains. A gene, *naaA*, was identified in the genome of strain P156 by sequence alignment. NaaA showed low amino acid sequence identity with functionally reported nicotinamidase. It showed the highest sequence identity (27.2%) with nicotinamidase from *Arabidopsis thaliana* (Fig. 6a). To confirm the function of *naaA*, it was cloned into pET28a, expressed and the His-tagged enzyme was purified. NaaA could transform nicotinamide into nicotinic acid without adding other coenzyme (Fig. 6b). The optimum pH and temperature for NaaA catalyzed reaction were 7.0 and 40 °C, respectively (Fig. 6c,d).

### The *naaD* gene encodes the 2,5-DHP 5,6-dioxygenase

**Figure 7:**
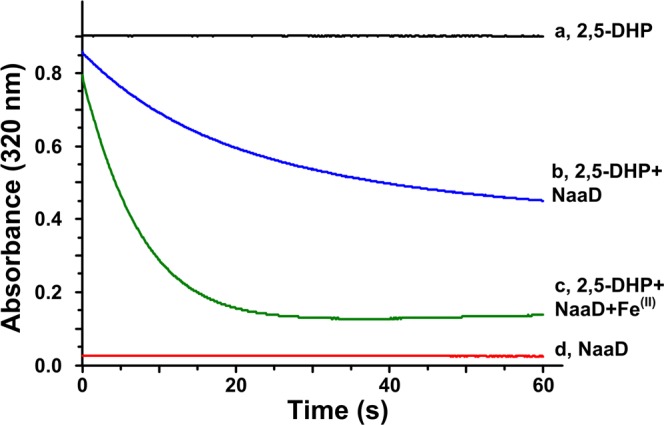
Characterization of NaaD. The enzyme activity was measured according to the absorbance decrease at 320 nm. The reaction mixture contain NaaD, 250 μM 2,5-DHP, and 5 μM FeSO_4_. (**a**) Negative control, 2,5-DHP; (**b**) reaction of NaaD (NaaD + 2,5-DHP); (**c**) reaction of NaaD (NaaD + 2,5-DHP + Fe^2+^); (**d**) negative control, NaaD.

The ring-cleavage reaction from 2,5-DHP to *N*-formylmaleamic acid is one of the key step in pyridinic compounds degradation, and this is due to that 2,5-DHP is the key metabolic intermediate of many pyridine derivatives^7,11,15,16,18^. To confirm its function, the *naaD* gene was cloned, heterologously expressed in *E. coli* cells and purified as a homogeneity form. The molecular mass of the purified band on SDS-PAGE was approximately ~40 kDa. Oxidative activity of the purified NaaD was measured at 320 nm, and the results indicated that NaaD is responsible for the 2,5-DHP conversion to form *N*-formylmaleamic acid (Fig. 7). The metal cheater binding amino acids are conserved among the aligned sequences (Fig. S3).The addition of Fe^2+^ significantly increased the enzyme activity of NaaD, indicating that Fe^2+^ is required for the oxidative activity of NaaD.

### The *naaE* and *naaF* genes are responsible for the conversion from *N*-formylmaleamic acid to maleic acid

**Figure 8:**
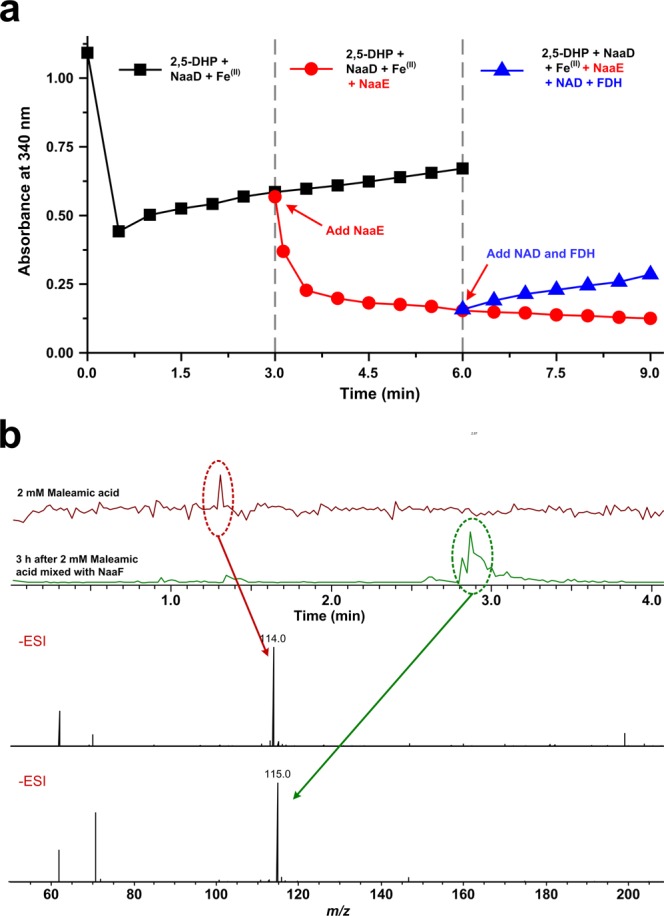
Characterization of NaaE and NaaF. (**a**) Enzymatic activity of NaaE. The enzyme activity was measured according to the absorbance change at 340 nm. *N*-formylmaleamic acid was produced by adding NaaD, 500 μM 2,5-DHP, and 5 μM FeSO_4_. NaaE activity was measured by adding NaaE to the NaaD catalyzed reaction mixture after 3 min. Formic acid was detected by adding 500 μM NAD^+^ and 0.5 unit formic acid dehydrogenase to the NaaE catalyzed reaction mixture. (**b**) LC-MS analysis of NaaF catalyzed reaction. 2 mM maleamic acid was mixed with NaaF and incubated at 25 °C for 3 hours.

Two genes (*naaE* and *naaF*), which are located on each side of the *naaD* gene, are predicted for the conversion from *N*-formylmaleamic acid to maleic acid. To confirm the function of these two genes, *naaE* and *naaF* was cloned and heterologously expressed in *E. coli* cells. As shown in Fig. 8a, the product of NaaD catalyzed reaction was used as the substrate for NaaE. The pyridine ring of 2,5-DHP was opened to form *N*-formylmaleamic acid within 30 seconds, and the absorbance at 340 nm gradually increased in the following 5.5 min. When NaaE was added at 3 min, the absorbance at 340 nm decreased indicating that *N*-formylmaleamic acid was converted by NaaE. To identified the product of NaaE catalyzed reaction, formate dehydrogenase and NAD^+^ were added 3 min after adding NaaE. The absorbance at 340 nm increased, indicating that formic acid was the product of NaaE catalyzed reaction (Fig. 8a). NaaE is a *N*-formylmaleamate deformylase, which transforms *N*-formylmaleamic acid to maleamic acid and formic acid.

To determine the function of NaaF, it was mixed with maleamic acid. After 3 h incubation, the product was detected by LC-MS. The result indicated that the signal representing maleamic acid (*m/z* of 114.0) disappeared, while a new signal with *m/z* of 115.0 was observed (Fig. 8b). The results indicated that maleamic acid was transformed into maleic acid by NaaF. The gene *naaF* encodes a maleamate amidase.

## Discussion

Pyridinic compounds have significant biological activities, the substitution groups on pyridine ring play important roles for their biological physiology^19^. The production of pyridine derivatives by organic synthesis requires strict conditions for high yield, resulting in high cost^20,21^. Biocatalysis, with high selectivity and mild reaction conditions, is a useful supplementary technology for the chemical industry in these reactions^22,23^. However, biocatalysis technology in pyridinic compounds production is limited by the lack of enzymes. Microorganisms, which are able to degrade pyridine and its derivatives, are important bio-resources to provide useful elements for biocatalysis of pyridinic compounds^22^. In this study, *Alcaligenes* sp. strain P156, which could use nicotinamide as its sole source of carbon, nitrogen and energy, was isolated and characterized. The first step of nicotinamide degradation in strain P156 is deamination to form nicotinic acid (Fig. 5). The following degradation pathway of nicotinic acid is similar with that in *P. putida* KT2440. Microbial degradation of nicotinamide has been reported previously, of which nicotinamide was transformed into nicotinic acid^12,13^. However, the further degradation of nicotinic acid in these microorganisms has not been reported. In this study, we revealed that strain P156 could transform nicotinamide to nicotinic acid, and nicotinic acid could be further degraded through 2,5-DHP pathway. For the first time, the complete nicotinamide degradation pathway is reported in one bacterium.

Nicotinamidases are nearly ubiquitous enzymes that convert nicotinamide to nicotinic acid in salvage pathway to produce NAD^+^, an important redox carriers in metabolism^24,25^. The roles of nicotinamidase in NAD^+^ biosynthesis, lifespan extension, germination, virulence, and eliminating inhibition, have been reported^24,26–29^. However, the role of nicotinamidase in growth with nicotinamide as the substrate has not been reported. Obviously, for most of these organisms, growth with nicotinamide is not the physiological role of nicotinamidase. For example, *E. coli* BL21 and *P. putida* S16 could transform nicotinamide into nicotinic acid. However, they could not grow with nicotinamide (Fig. S4). Interesting that strain *P. putida* KT2440 could grow well with nicotinic acid^11^; however, it grew weak with nicotinamide (OD_600 nm_ = 0.10 after 72 h cultivation) (Fig. S4). The results indicated that although nicotinamidase was expressed and worked in these strains, the expression level was too low to support the growth of these strains with nicotinamide. Unlike strain KT2440, strain P156 grew better with nicotinamide than with nicotinic acid (Fig. S4). Growth kenetics study revealed that nicotinamide has no inhibitory effect on the growth of strain P156 at concentration range from 0 to 3 mg/mL, therefore, it can be used as growth substrate for bacteria in the environment. Nicotinamide degradation for growth purpose is one of the physiological roles of nicotinamidase. We have compared all the *Alcaligenes* from GenBank database with the nicotinamide degradation genes in P156, it turned out that the nicotinic acid degradation cluster are highly conserved in all of these 30 strains. However, 9 of these strains do not have the NaaA gene. The results indicated that nicotinic acid degradation cluster is belong to the core genome of *Alcaligenes*, however, nicotinamidase is belong to the variable genome. The *naaA* gene was introduced into the genome of P156 and give the capacity of growing with nicotinamide for strain P156.

Several pyridine ring *α*-hydroxylases have been reported, all of them are molybdate containing enzymes including nicotinate dehydrogenase in *P. putida* KT2440^11^. This kind of hydroxylases contain a large subunit with molybdenum-containing domain, a small subunit with [2Fe-2S] cluster-binding domains, and some of them also have a middle FAD-binding subunit. In spite of the middle subunit, all the reported enzymes has only one molybdenum-containing large subunit. However, in *A. faecalis* JQ135 Zhang *et al*. has proved that the nicotinate dehydrogenase is a three components hydroxylase, including two molybdenum-containing subunit^30^. The *alfp_2914* to *alfp_2916* genes show high sequence identity with the related genes in strain JQ135, the function of these *orf*s involved in nicotinate transformation should be further confirmed. AlFP_1623 has the highest sequence identity with 6-hydroxynicotinate 3-monooxygenase in strain KT2440. We cloned this gene and purified the His-tagged enzyme; however, enzyme activity was not detected. Therefore, further investigation should be done to elucidate the complete molecular mechanism of nicotinamide degradation.

## Methods

### Chemicals and media

Nicotinamide, nicotinic acid, 6-hydroxynicotinic acid and other pyridinic compounds were purchased from Aladdin (Shanghai, China). 2,5-DHP was purchased from SynChem OHG (Kassel Corp., Kassel, Germany). FAD, NADH were obtained from Sigma-aldrich company. All other reagents and solvents were of analytical or chromatographic grade and were commercially available. The mineral salt medium (MSM) was used for enrichments and isolation as previously described^31^. Soluble pyridinic compounds were added into MSM before inoculation from aqueous stock solutions after sterilized by 0.22 μM filtration.

### Strain and cultivation conditions

The enrichment cultivation was carried out by adding 5 g contaminated soil from a solid waste treatment plant (Qingdao, China) into 50 mL MSM with 1 mg/mL PIA. The mixture was cultivated aerobically in a shaker at 30 °C 120 rpm for a week. The culture was then transferred into new MSM every other week. Domestication processes lasted for 2 months before strain P156 was isolated using several times of streaking inoculations. The 16S rRNA gene of strain P156 was amplified using universal primers 27F (5′-GAGTTTGATCATGGCTCAG-3′) and 1492R (5′-GGTTACCTTGTTACGATC-3′). PCR amplification was performed with 30 μL reaction mixtures containing primers, genome of strain P156 and 15 μL 2 × Taq Mix (Novoprotein). PCR was carried out as follows: 5 min at 95 °C and then 35 cycles of 30 s at 95 °C, 30 s at 60 °C, and 90 s at 72 °C. The PCR product was sequenced in Sangon Biotech (Shanghai). Sequences alignment and phylogenetic inferences were performed using Clustal W, and the tree was constructed by MEGA6 software obtained using the neighbor-joining method with a bootstrap of 1,000. The 16S rRNA gene sequences of typical species in *Alcaligenes* were obtained from list of prokaryotic names (LSPN) database.

### Substrates specificity for growth and biotransformation of strain P156

Different substrates were added to MSM at a final concentration of 1 mg/mL, and the cultures were incubated at 30 °C for 72 hours. The growth of cultures was detected according to OD_600 nm_. Resting cell reactions were performed to identified the transformation of different pyridine derivatives by strain P156. Cells cultivated with PIA in the late exponential phase were centrifuged at 8,000 × g for 15 min at 4 °C. The pellet was washed twice with 50 mM PBS buffer (pH 7.0), and resuspended in the same buffer to OD_600 nm_ = 5.0. The bacterial suspension was designated as resting cell and used immediately in the biotransformation experiments. The experiments were conducted using a series of 250 mL Erlenmeyer flasks, each containing 25 mL resting cell and 0.5 mg/mL substrate. Samples were collected at interval times and centrifuged. Then the supernatants were analyzed by UV-visible spectrophotometer.

### Growth kenetics study

In a batch system, the specific growth rate (*μ*) was defined as

1 $$\frac{dX}{dt}=\mu X-{K}_{d}X$$

*X* is cell concentration (OD600 nm), *μ* is specific growth rate of bacteria (h^−1^), and *K*_*d*_ is endogenous decay coefficient (h^−1^). At exponential growth phase the *K*_*d*_ could be neglected, and the equation (1) was reduced to

2 $$\frac{dX}{dt}=\mu X$$

The value of *μ* was determined at the exponential phase of the growth curve according to equation (2).

Two models were used to fit to the experimental data obtained from the batch experiments. Monod model represents bacterial growth under substrate-limited and non-inhibitory conditions as equation (3).

3 $$\mu =\frac{{\mu }_{max}{\rm{S}}}{{K}_{s}+{\rm{S}}}$$

Haldane inhibitory growth model represents the growth kinetics of an inhibitory compound such as pollutants^32,33^ as equation (4)

4 $$\mu =\frac{{\mu }_{app}\ast {S}_{0}}{{K}_{s}+{S}_{0}+(\frac{{{S}_{0}}^{2}}{{K}_{i}})}$$

The values of the kinetic parameters were obtained from non-linear fitting using Origin software.

### Intermediates identification

Resting cell reactions were performed with 1 mg/mL nicotinamide at 30 °C 120 rpm. Samples were collected at interval times and centrifuged. Fractions of the supernatant were diluted 10 times with methanol, and analyzed by HPLC and LC-MS.

### Cloning, expression and purification of four genes

Four gene, *naaA*, *naaD*, *naaE* and *naaF*, were amplified from genomic DNA of strain P156, using primers shown in Table 2. The PCR product was purified and inserted between the restriction sites of expression vector pET28a containing a *N*-terminal His_6_-tag. The recombinant plasmids were verified by sequencing and transformed into *E. coli* BL21(DE3) cells for recombinant expression, respectively. Each *E. coli* BL21(DE3) cells carrying recombinant plasmid was grown in LB medium with 50 μg/mL kanamycin at 37 °C to an OD_600 nm_ of 0.6~0.8. Protein expression was induced by adding 0.5 mM isopropyl β-D-1-thiogalactopyranoside (IPTG), and the culture was then shaken at 30 °C overnight. Cells were harvested by centrifugation, dissolved in 50 mM Tris-HCl (pH 8.0) buffer, and broken by ultrasonication. His-tagged enzymes were purified in a gravity wash column with Ni^2+^-NTA His Sefinose resin (Sagon Biotech, Shanghai). The resin was balanced in 50 mM Tris-HCl (pH 8.0) buffer, and the protein was washed by the same buffer with 50 mM imidazole.

**Table 2:** Oligonucleotides used in this study.

Name	Sequence (5′-3′) (restriction sites are underlined)
*naaA*-F	**CGCGGATCC**ATGGCTATTCAGATTGAT
*naaA*-R	CCG**CTCGAG**TTATTGTCTGGATTTCTG
*naaD*-F	CGC**GGATCC**ATGGCAGTTAGTGATTATC
*naaD*-R	CCG**CTCGAG**TCATTCGTACTCACCAACG
*naaE*-F	CGC**GGATCC**ATGAGTACCTTTCTTTACG
*naaE*-R	CCG**CTCGAG**TCACACCAGGCGTTGGCCC
*naaF*-F	**CGCGGATCCTTG**GAGCCTAAAGCTCCT
*naaF*-R	CCG**CTCGAG**TTAAACTCCAATCAACTT

PCR primers *naaA*-F/-R, *naaD*-F/-R, *naaE*-F/-R and *naaF*-F/-R are used for the construction of expression recombination plasmid pET-28a-*naaA*, pET28a-*naaD*, pET28a-*naaE*, and pET28a-*naaF*.

### Enzyme assay

The nicotinamidase activity was monitored according to the decrease of substrate nicotinamide. The reaction was terminated by adding 0.1 volume of 1M H_2_SO_4_, and, after 3 min, 0.2 volume of 1M NaOH and 0.7 volume of methanol were added. After that, the substrate concentration in the reaction mixture was analyzed by HPLC. The 2,5-DHP dioxygenase activity was monitored according to the absorbance at 320 nm in 50 mM Tris-HCl buffer (pH 8.0) at room temperature (25 °C) in UV2600 UV-Vis spectrophotometer (Shimadzu, Japan). The cuvette contained NaaD and 250 μM 2,5-DHP in the presence or absence of 5 μM FeSO_4_. The 6-hydroxynictinic acid 3-monooxygenase activity was determined by measuring the decrease in absorbance at 340 nm due to the substrate-dependent oxidation of NADH. Assays were carried out in 50 mM Tris-HCl buffer (pH 8.0) at room temperature. The cuvette contained cell extract of strain P156, 250 μM substrate and 250 μM NADH in a total volume of 800 μL. The enzyme assays were initiated by the addition of enzyme.

### Analytical methods

Pyridinic compounds were analyzed by TLC, HPLC (Agilent, series 1100) or UV2600 UV-Vis spectrophotometer. The samples were subjected to TLC analysis using silica gel 60 F254. The composition of the eluent was chloroform:methanol:acetic acid = 10:1:0.1 (vol/vol/vol). After the separation process, the plate was dried and observed at 254 nm. The pyridinic compounds in resting cell reactions were determined by HPLC using an Eclipse XBD-C_18_ reverse-phase column (5 μm; 4.6 × 150 mm; Keystone Scientific, Bellefonte, PA) with a DAD detector. The mobile phase consisted of 85:15 (vol/vol) methanol: 1 mM H_2_SO_4_ at a flow rate of 0.5 mL/min at 30 °C. The mobile phase for the samples of NaaA catalyzed reaction consisted of 80% (vol/vol) 20 mM ammonium acetate and 20% (vol/vol) methanol at a flow rate of 1.0 mL/min at 30 °C. LC-MS analysis was performed on an Agilent 6460 triple quadrupole system equipped with electrospray ionization (ESI) sources in 20% (vol/vol) methanol and 80% (vol/vol) deionized water (18 MΩ/cm) (0.05% formic acid [vol/vol]) at a flow rate of 0.2 mL/min with the same Eclipse XBD-C_18_ reverse-phase column. All samples were treated with the addition of 9 volumes of methanol at 4 °C for 10 min followed a centrifugation at 12,000 × g for 2 min. Then the samples were filtered through a 0.22-μm Sartorius filter prior to HPLC and LC-MS analysis.

### Nucleotide sequence accession numbers

This Whole Genome Shotgun project has been deposited at DDBJ/ENA/GenBank under the accession CP021079. The sequences of 16S rRNA gene from strain P156 is available in GenBank under accession numbers KU740245.

### Ethical statement

This article does not contain any studies with human participants or animals performed by any of the authors

## Supplementary information

Suppnementary information

## References

[1] Maga JA (1981). Pyridines in foods. J Agr Food Chem.

[2] O’Hagan D (2000). Pyrrole, pyrrolidine, pyridine, piperidine and tropane alkaloids. Nat Prod Rep.

[3] Scriven, E. F. & Murugan, R. Pyridine and pyridine derivatives Kirk-OthmerEncyclopedia of Chemical Technology 4th edn, Vol 20 John Wiley & Sons, Inc: Hoboken, NJ. (2005).

[4] Sims GK, O’Loughlin EJ, Crawford RL (1989). Degradation of pyridines in the environment. Crit Rev Environ Control.

[5] Richards DJ, Shieh WK (1986). Biological fate of organic priority pollutants in the aquatic environment. Water Res.

[6] Kuhn EP, Suflita JM (1989). Microbial degradation of nitrogen, oxygen and sulfur heterocyclic compounds under anaerobic conditions: studies with aquifer samples. Environ Toxicol Chem.

[7] Kaiser JP, Feng Y, Bollag. JM (1996). Microbial metabolism of pyridine, quinoline, acridine, and their derivatives under aerobic and anaerobic conditions. Microbiol Rev.

[8] Zalat OA, Elsayed MA (2013). A study on microwave removal of pyridine from wastewater. Engineering.

[9] Agrios A, Pichat P (2006). Recombination rate of photogenerated charges versus surface area: Opposing effects of TiO_2_ sintering temperature on photocatalytic removal of phenol, anisole, and pyridine in water. Journal of Potochemistry and Photobiology A:Chemistry.

[10] Fetzner S (1998). Bacterial degradation of pyridine, indole, quinoline, and their derivatives under different redox conditions. Appl Microbiol Biotechnol.

[11] Jimenez JI (2008). Deciphering the genetic determinants for aerobic nicotinic acid degradation: the nic cluster from *Pseudomonas putida* KT2440. Proc Natl Acad Sci USA.

[12] Hughes DE, Williamson DH (1953). The deamidation of nicotinamide by bacteria. Biochem J.

[13] Gadd REA, Johnson WJ (1974). Kinetic studies of nicotinamide deamidase from *Micrococcus lysodeikticus*. International Journal of Biochemistry.

[14] Bogan LK, Brenner C (2008). Nicotinic acid, nicotinamide, and nicotinamide riboside: A molecular evaluation of NAD^+^ precursor vitamins in human nutrition. Annual Review of Nutrition.

[15] Orpin CG, Knight M, Evans WC (1972). The bacterial oxidation of picolinamide, a photolytic product of diquat. Biochem J.

[16] Tang H (2013). Systematic unraveling of the unsolved pathway of nicotine degradation in *Pseudomonas*. PloS Genet.

[17] Yu H (2011). Complete genome sequence of the nicotine-degrading *Pseudomonas putida* strain S16. Journal of bacteriology.

[18] Shukla OP, Kaul SM (1986). Microbiological transformation of pyridine *N*-oxide and pyridine by *Nocardia* sp. Can J Microbiol.

[19] Kiener A, CGlockler R, Heinzmann K (1993). Preparation of 6-oxo-1, 6-dihydropyridine-2-carboxylic acid by microbial hydroxylation of pyridine-2-carboxylic acid. J Chem Soc Perkin Trans.

[20] Hill MD (2010). Recent strategies for the synthesis of pyridine derivatives. Chemistry.

[21] Movassaghi M, Hill MD, Ahmad OK (2007). Direct synthesis of pyridine derivatives. J Am Chem Soc.

[22] Yu H, Tang H, Xu P (2014). Green strategy from waste to value-added-chemical production: efficient biosynthesis of 6-hydroxy-3-succinoyl-pyridine by an engineered biocatalyst. Sci Rep.

[23] Schmid A (2001). Industrial biocatalysis today and tomorrow. Nature.

[24] Wang G, Pichersky E (2007). Nicotinamidase participates in the salvage pathway of NAD biosynthesis in *Arabidopsis*. The Plant journal: for cell and molecular biology.

[25] Stekhanova TN (2014). Nicotinamidase from the thermophilic archaeon *Acidilobus saccharovorans*: structural and functional characteristics. Biochemistry. Biokhimiia.

[26] van der Horst A, Schavemaker JM (2007). Pellis-van Berkel, W. & Burgering, B. M. The *Caenorhabditis elegans* nicotinamidase PNC-1 enhances survival. Mechanisms of ageing and development.

[27] Hunt L, Holdsworth MJ, Gray JE (2007). Nicotinamidase activity is important for germination. The Plant journal: for cell and molecular biology.

[28] Kim S (2004). *Brucella abortus* nicotinamidase (PncA) contributes to its intracellular replication and infectivity in mice. FEMS microbiology letters.

[29] Anderson RM, Bitterman KJ, Wood JG, Medvedik O, Sinclair DA (2003). Nicotinamide and PNC1 govern lifespan extension by calorie restriction in *Saccharomyces cerevisiae*. Nature.

[30] Zhang Y (2018). Complete genome sequence of *Alcaligenes faecalis* strain JQ135, a bacterium capable of efficiently degrading nicotinic acid. Current microbiology.

[31] Yu H, Zhao S, Lu W, Wang W, Guo L (2018). A novel gene, encoding 3-aminobenzoate 6-monooxygenase, involved in 3-aminobenzoate degradation in *Comamonas* sp. strain QT12. Applied microbiology and biotechnology.

[32] Kumar A, Kumar S, Kumar S (2005). Biodegradation kinetics of phenol and catechol using *Pseudomonas putida* MTCC 1194. Biochemical Engineering Journal.

[33] Park C, Kim TH, Kim S, Lee J, Kim SW (2002). Biokinetic parameter estimation for degradation of 2,4,6-trinitrotoluene (TNT) with *Pseudomonas putida* KP-T201. Journal of bioscience and bioengineering.

