# Supplementary material for: Microbial Degradation of Nicotinamide by a Strain Alcaligenes sp. P156
Source: Sci Rep. 2019 Mar 6;9:3647. doi: 10.1038/s41598-019-40199-0 (PMC6403243; doi:10.1038/s41598-019-40199-0)

Supporting Information

Microbial Degradation of Nicotinamide by a Strain *Alcaligenes* sp. P156

Chunhui Hu[1,2†], Shuxue Zhao[1†], Kuiran Li[2*], Hao Yu[1*]

[1]Shandong Provincial Key Laboratory of Applied Mycology, College of Life Sciences, Qingdao Agricultural University, Qingdao, People's Republic of China

[2]College of Marine Life Science, Ocean University of China, Qingdao, People's Republic of China

†Chunhui Hu and Shuxue Zhao contributed equally to this work.

*To whom correspondence should be addressed:

Hao Yu, E-mail: yuhaosunshine@163.com.

700 Changcheng Road, Chengyang District, Qingdao, Shandong Province, People's Republic of China, Tel: +86-532-88030292

Kuiran Li E-mail: likr@ouc.edu.cn

238 Songling Road, Laoshan District, Qingdao, Shandong Province, People's Republic of China, Tel: +86-532-66782758

Keywords: nicotinamide, biodegradation, growth kinetics, gene cluster, *Alcaligenes*

S1 Figure

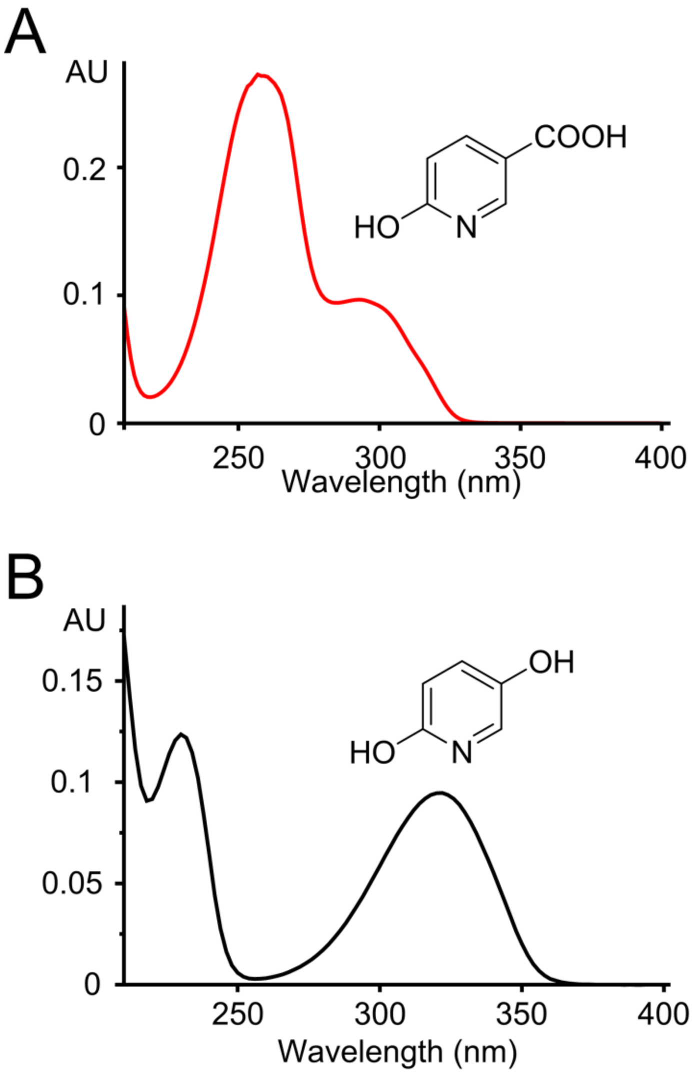

S1 Figure Spectra of 6-hydroxnicotinic acid and 2,5-dihydroxypyridine in Figure 4a.

S2 Figure

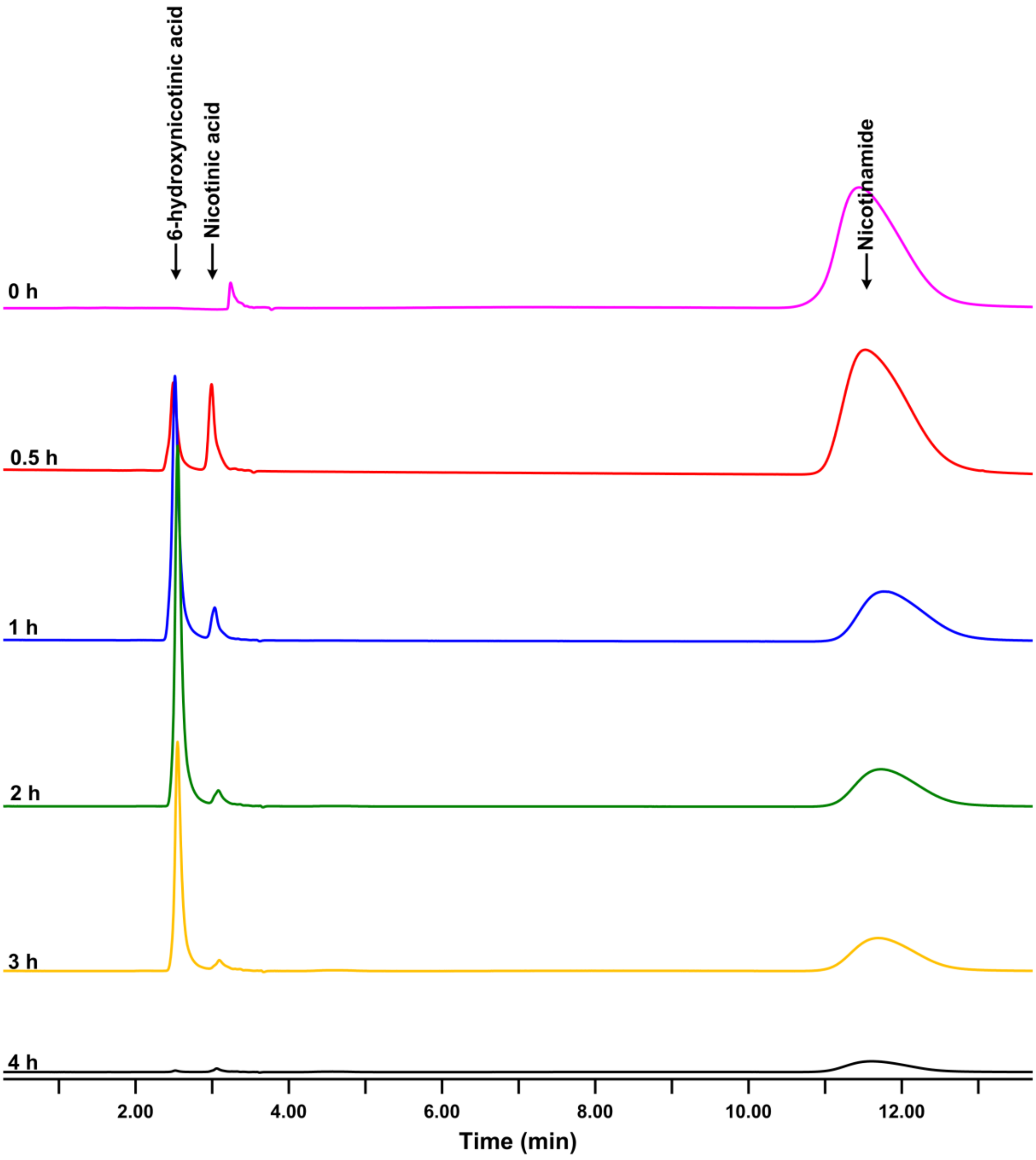

S2 Figure HPLC analysis of nicotinamide degradation by *Alcaligenes* sp. P156. HPLC was performed with a Waters Spherisorb ODS2 C18 reverse phase column. The mobile phase consisted of 80% (vol/vol) 20 mM ammonium acetate and 20% (vol/vol) methanol at a flow rate of 1.0 mL/min at 30 ℃.

S3 Figure

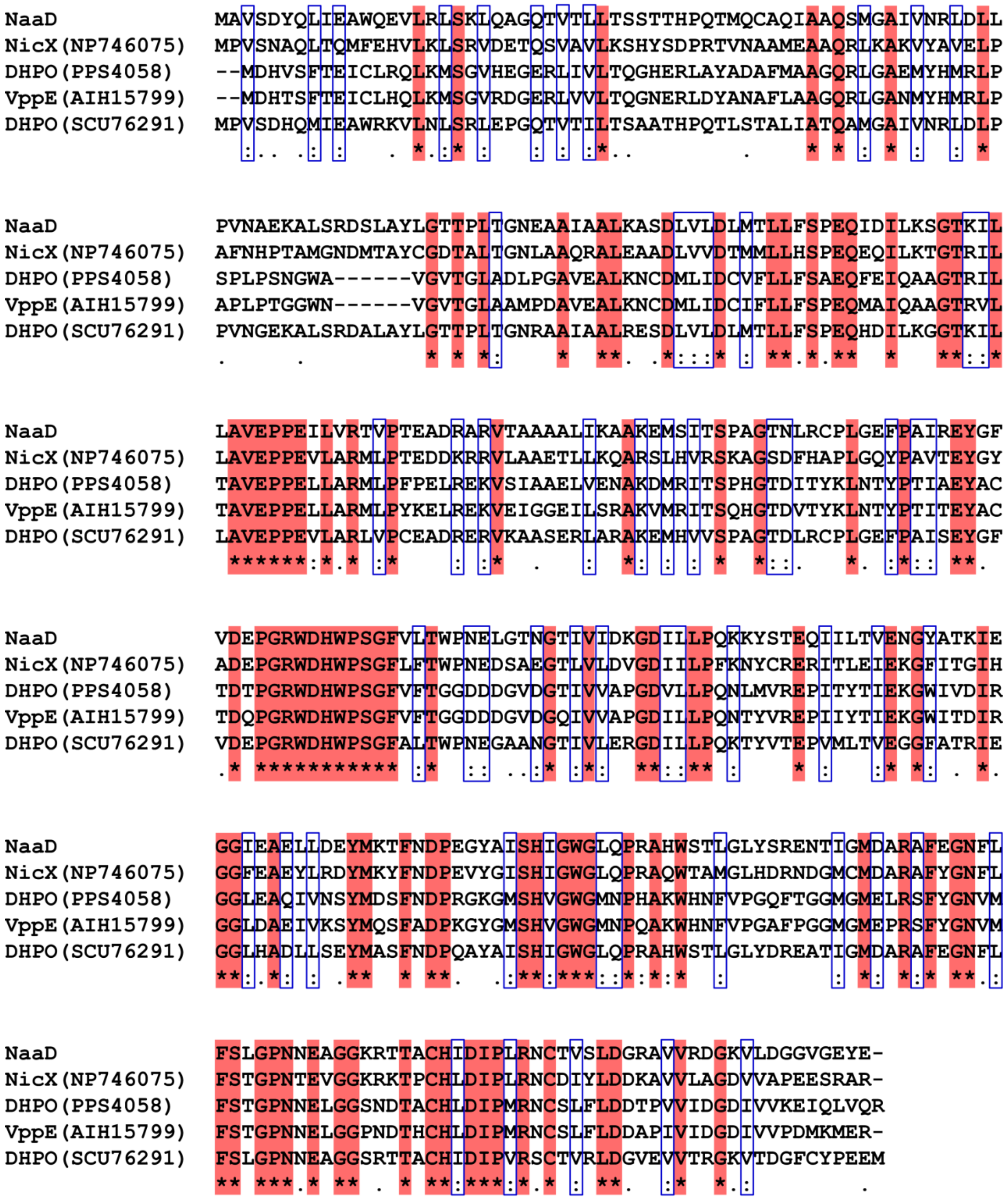

S3 Figure Sequence alignment of NaaD with the homologous proteins.

S4 Figure

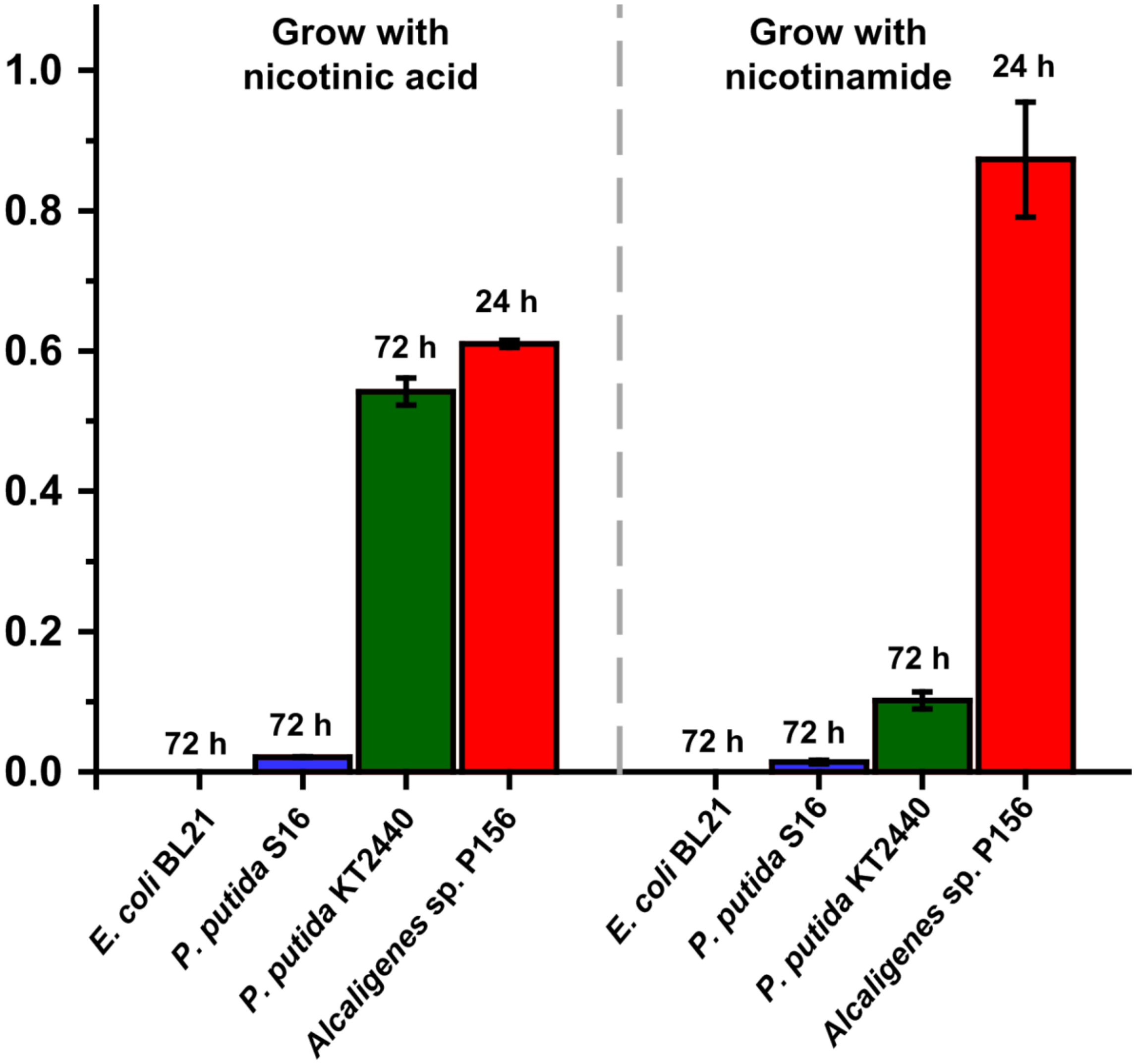

S4 Figure Strain P156, *E. coli* BL21, *P. putida* S16 and *P. putida* KT2440 that were cultivated in MSM medium with nicotinic acid or nicotinamide as the substrate. The cultivation time was indicated on top of the bar.

Sequence of predicted genes involved in nicotinamide degradation

>*naaA*

ATGGCTATTCAGATTGATCAAATCAATCCCGGCAGCACGGCGGTCATTGTGATCGAC
ATGCAAAACGACTTCATCGCCCCCGGCGCCCCGCTGGAAACACCCATGGGCATGGA
GCTGATGCCCCGCCTGCAAAAACTGCTGGGCCATGCCCGCCAAACCGGCATGGAAG
TGATCTTCACCGCCCACGCCCACCGCCGCAACGGCTGCGATATGGGCCTGTTCGGG
GAGATTTACCCTCCGATTCAAAACCAGGTGGGACTGGTCGATGAGACCGCTGGCGT
TGATATCTACCCCGAAGTGGCGCCACAAAGGGACGAGGTCGTCATCAAAAAGCATC
GGTACAGCGCGTTCTTTGGCACAGACCTGGACATCATCCTGCGTACCCGCAGGATCG
ATACGGTTGTGATCACCGGCGTGACCACTGAGAACTGCTGCCACGCCACGGCCCGC
GATGCCATGTTCCATGGCTACAAAGTGGCTTTCATCTGCGATGCGACCGGCACCTAC
AACTACCCGGATATGGGCTTTGGGACGATTCCCGCTGAAGAAGTGCACCGCGTCAC
CCTGGGAGTGCTGGCGGTATCCACCGCTCATGTCATGACGACAGATGAGCTGATTCA
GAAATCCAGACAATAA

>*alfp_1623*

ATGGAAAAAACAGTCAAGATTGCGATTGTAGGGGCCGGTCTTGGCGGAGCCGCTGC
GGCGACTCTGTTGCAGCAAGCTGGCTTTGAGGTAGAAGTATTTGAACAGGCGCCAG
AATTCTCGCGTTTGGGTGCCGGTATCCACGTGGGCCCCAACGTGATGAAGATTTTCC
GCCGCATGGGTCTGGAAAAAGCACTGGACGATATGGGTTCGCACCCTGATTTCTGGT
TCAGCCGTGATGGCGAAACGGGCGACTACCTGTCCCGTATCCCTCTGGGTGAATTTG
CCAAGAAAGAATACGGTGCGTCGTACATCACTGTGCACCGTGGCGATATGCACGCCT
TGCAAATTGACAGCATCAAGCCGGGCACCGTGCACTTTGGCAAGCGTTTGCAAAAC
CTGGTGGATCGTGGTGACGACGTCTTGCTGGAATTTGCCGATGGCACCAGCGTGAC

GGCTGACATTGTGATTGGTGCCGATGGCATTCACTCCAAGATCCGCGAAACCCTGCT
GGGCGTAGAAGACCCGATTTACAGCGGCTGGGTGGCACACCGTGCCCTGATCCGTG
GCGAAAATCTGGCTCGCTTTGCCGACGAGTTCGAGGACTGTGTGAAATGGTGGACT
GACGACCGTCACATGATGGTGTACTACACCACCGGCAAGCGCGATGAATACTACTTT
GTGACTGGCGTGCCACATCCTGCCTGGGATTTCCAGGGTGCGTACGTAGACAGCTC
GCAAGAAGAGATGCTGGCCGCATTTGAAGGCTATCATTCCACGGTTCAGAACCTGAT
CAAATCGACCGAAAGCATTACCAAGTGGCCATTGCGCAACCGCAATCCGCTGCCTTT
GTGGAGCCGTGGTCGTCTGGTCATGTTGGGCGATGCATGTCACCCCATGAAGCCAC
ACATGGCTCAAGGTGCTTGCATGGCTATTGAAGATGCCGCCATGTTGACTCGCTGCT
TGCAAGAGACCGGTTTGTCGGATTTCCGCACGGCCTTTGATCTGTACGAGCTCAACC
GCAAAGAGCGCGCGACTCGTGTCCAGTCCGTGTCCAACGCCAACACCTTCTTGCGC
ACGCAAGAAGATCCGGCCTGGGTTTACGGCTACGACCTGTACGGTCAGGACCTGAA
GTCCAGCTAA

> *alfp_naaF*

TTGGAGCCTAAAGCTCCTTACGGTTTGTTGATTATTGATCTGGTGAATGGCTTTGCTG
ACCCGGCGGTGTTTGGCGGCGGCAATATTCCTGAAGCGATCGAGAACACGCAGAAG
TTGTTGGCGACGGCGCGTGAACAAGGCTGGCCGGTTGCTCATACCCGTATCGTGTAT
GCGGACGATGGCGCTGATAACAACATCTTCAGCATCAAAGTCCCTGGCATGCTGGG
CCTGACCGAAGACGCGCACAACAGCCACATCGTGCCGGAGTTGGCTCCTGCTCCCG
GGGAGTTCGTTGTTCGCAAGAATGTGCCGTCGGCCTTCTTTGGCACGCCCTTGGCG
GCGTGGCTGACCCAGCGTGGTGTGCAAACACTGCTGGTCGCTGGTGCGGTTACCAG
TGGTTGCGTGCGTTCCAGCGTGGTCGATGCGATGCAACTGGGCTTTCGCCCCCTGGT
CGTCTCTGATTGCGTGGGTGACCGTGCGATTGGCCCACACGAGGCTAACCTGTTCG

ACATGCGACAGAAGTACGCCACCGTGGCAACGCGTGAAGAGGCGATGAAGTTGATT
GGAGTTTAA
>*naaD*
ATGGCAGTTAGTGATTATCAACTTATCGAGGCATGGCAAGAAGTGCTGCGCCTGTCC
AAGCTGCAAGCGGGTCAAACCGTGACCTTGCTGACCAGCTCGACCACTCACCCGCA
GACCATGCAGTGCGCGCAGATTGCCGCTCAGTCCATGGGCGCGATTGTGAATCGCTT
GGACTTGTTGCCGGTCAACGCGGAAAAAGCCTTGAGCCGTGACTCGCTGGCGTACT
TGGGTACGACCCCGCTGACGGGTAACGAAGCGGCGATCGCAGCCTTGAAGGCCAG
TGATCTGGTTCTGGACCTGATGACCCTGTTGTTCTCGCCCGAGCAGATCGACATTCT
GAAGTCGGGCACCAAGATCCTCTTGGCGGTGGAGCCACCCGAAATTCTGGTTCGTA
CTGTGCCAACCGAAGCAGATCGCGCACGCGTGACCGCTGCGGCCGCTTTGATCAAG
GCCGCCAAGGAAATGAGCATCACCTCTCCTGCCGGCACTAATCTGCGTTGCCCGCTG
GGTGAATTCCCTGCGATTCGTGAATACGGTTTTGTGGATGAACCCGGTCGTTGGGAC
CACTGGCCCAGCGGTTTCGTGCTGACCTGGCCTAATGAATTGGGTACCAACGGCAC
CATTGTGATCGACAAGGGCGACATCCTGTTGCCACAGAAGAAATACTCGACCGAGC
AGATCATTTTGACCGTGGAAAACGGCTACGCCACCAAGATTGAAGGTGGTATCGAA
GCCGAGCTGCTGGATGAATACATGAAGACCTTCAACGATCCCGAAGGTTATGCAATT
TCCCACATTGGTTGGGGTTTGCAGCCACGTGCTCACTGGTCCACGCTGGGGCTGTAC
TCGCGTGAGAACACCATTGGTATGGATGCTCGTGCGTTCGAGGGTAACTTCCTGTTC
TCCCTGGGCCCGAACAATGAAGCTGGTGGCAAGCGTACAACGGCATGCCACATTGA
TATTCCTTTGCGCAATTGCACGGTCAGCCTGGATGGCCGCGCCGTAGTGCGTGATGG
CAAAGTACTAGACGGAGGCGTTGGTGAGTACGAATGA
>*naaE*

ATGAGTACCTTTCTTTACGGCGGCCATGTTCACGCCAATGGCATTCGTCAGCATTACC
TGCGTTACGGTGGTTCCAGCGAGGGTCGCGATCAGCGCCCAGCGGTCATCATCGTTC
CCGGCATTACCAGCCCGGCGGTGACCTGGGGTTTTGTGGGCGAGCAGTTCGGCAAG
CACTTTGATACCTACATTCAGGACGTGCGTGGTCGCGGTTTGAGCGAAGCGGCTGA
AGGCATGGATTACAGCCTGGATGCGCAAGCCGATGACTTGATCGCCTTGGCTCAAGC
CTTGGGTCTGAAAGACTACATCGTGGTCGGCCACTCCATGGGTGCACGTATAGGTTT
GCGTGCGGCTCACAAGAATAGCGATGGCCTGAATCGTCTGGTGATGGTTGATCCTCC
GGTCTCTGGCCCAGGTCGTCGCGCTTATCCCTCCAAGTTGCCCTGGTATGTGGACTC
CATGGCCATGGCTCGCAAGGGTTGTACCGCAGAAGACATGCGTGCCTTTTGCCCGA
CCTGGACGGAAGAGCAATTGCAGTTGCGTGCTCAGTGGCTGCACACCTGCCACGAG
CCTGCGATTCTGGCCAGCTTCGAAGGTTTCCACACCGACGATATTCACGTCGACTTC
CCGCATCTGAAAGTACCTGCCTTGCTGATGACAGCCGAGCGTGGTGACGTGGTGCG
CGATGAAGATGTGGCCGAGATTCAGCAACTGGCCTCCGGCGTTCAGCACGTTCGTG
TACCGAACGCAGGTCACATGATTCCTTGGGATAACGAAGCAGGCTTCTACGAAGCTT
TCGGTGATTTCCTGGGCCAACGCCTGGTGTGA
>*alfp_2914*
ATGTCTTACGATGCGCCGCGCCACCAGCAGCAGGACTTGCTCAGGGCGTCCGGCAC
CTTGCTGATTTTAGCGGCACCGGCACGGCCTCCCAAGCCTTCGCCCGGCCAGCCCG
GCGTGGTGTCCGAGTACTTGCAGGCGCACGACGATGTCTTTGTCGCCGTGTCCGAA
CAGGGCTGGGTGCGCGCTTTTAACGGGCACGTGGACCTGGGCACCGGCATTCAAAC
TGCGCTAAGTCAGATCGTGGCCGACGAGCTGGATGTGCCCATGGCGCGCGTGCAAA
TGGTGCTGGGCCATACCGATGCTGTGCCCAATCAAGGTCCTACCATCGCCAGTGCGT
CCATTCAGATCCATGCAGTACCGCTGCGCAAGGCGGCGGCTCAGGCACGTCAATTA

CTGCTGGCACAAGCTGCAGAGCGCTGGACGGTGCCTGCCGATCAATTACGCGTGGA
AGACGGCACAATCATTGCCCCGGACGGGCGCAGCCTGACGTACTGGCAATTGCTGG
AAGGTTTGGAGTTGCGAGCCTATCTGGATAAGGAAACCCCCACCAAACCGGCCGAG
CAATTGCGCATTGTAGGGACAGCCCAAGCGCGTGTCGATATTCCGGGCAAGGTCGC
GGGGCAGTGGGTGTATGTACACGATGTGCGCGTGCCCGGCATGTTGCATGGTCGTGT
CGTGCGTCCTCCTTATATAGGGCGCGATAGCGGCGACTTCATTGGTCGCAGCCTGGA
ATCCGTGGACGAGCAGTCCATCCGTCATATTGCCGACGATGTGCGTGTCGTGGTGAT
TGGTGACTTTATTGGTGTGGTCGCGCGTCGTGAAGAACACGCCATGCGCGCCGCCC
GTGAATTGAACGTGCGCTGGAAAGCGATTCCTCCTTTGGAGGATATGAGTAATCTGG
AACAGCTGATTCGTCGTCAGCCCATGACGGAGCGCTTGCTGGCGGATAAAGGGCCG
GATTTTGATGAACTGCCGCCAGAGGGCAAACGCTTGAAGCGTACCTATGTCTGGCCT
TTTCAGCTGCATGCCTCCATTGGCCCGTCTTGCGCCGTGGCGGACTACCAGCCCGGC
CATAGCCGAATCTGGTCAGGCTCGCAGAATCCGCATATGTTGCGCGTCCACTTGAGT
CAGTTGCTGGACGAGGACGAAGCCGGCCTGGAGATCATCCGCCATGAAGCAGCCG
GTTGCTATGGCCGTAATTGTGCTGACGATGTATGTGCGGACGCCTTGCTCTTGTCCCG
GGCCGTCGGTGCGCCGGTACGTGTGCAACTGACGCGTGAACAAGAACACGGCTGG
GAGCCTAAAGGCGCTGCTCAGCTGATGGATGTGGAAGGCTCCATTGATGCCGAAGG
GCAGCTGCGTCATTACGATTTTGTGACCCATTACCCCTCCAACGATGCGCCGAACCT
GGCGCTCTTGCTGACGGGGCGGGAGTCGGCTGCTCCACGTCAATTGGAAATGGGGG
ATCGCACGGCAGTACCGCCGTACAGCTATCCCAAGCAGCGCATTGTGTGCCAGGAC
ATGCCTGCGATTGTGCGTGCCTCCTGGTTGCGCGGCGTGTCGGCCATGCCCAATTCT
TTTGCGCATGACTGCTTTCTGGACGAGCTGGCGGTGGAAGCAGGTGTAGACCCCTT
GAGTTATCGCCTGCGTTATCTGGATCAGGATGGACGCGCCCAGGAACTGCTGCAGG

CGGTGGCGGACAAGGGGCAATGGCAGATTGGGCATCGAGGTTCTCGCGGGCAGCC
GGACGAGCAAGGCTATCTCTACGGTCGTGGCTTGTCGTACGCCCGTTATATCCACAG
CAAGTTTCCTGGTTTCGGGGCGGCATGGTCAGCCTGGCTGCTGGATCTGCGGGTCCA
TGCGGAGACGGGCGTGATTGAGGTTCAGCAGATTCATGTGGGCCAGGACACCGGGC
AGATGGTGAACCCGGCGGGCGTGCGCCACCAGATTCACGGCAATGTGATCCAGTCC
TTGAGCCGCACTTTATATGAACAGGTGCGCTTTAACGCACAGGGTGTGGTCAGCGC
AGAGTGGGGCGCCTATCCGATTGTGGATTTCCCGCGCATCCCGCCCATTGAGGTGAT
TTTGATGGACAGGCAATCCGAGCCGCCGATGGGGTCGGGTGAATCTGCCTCTGTGC
CCTGTGCCTCGGCCATTGCCAATGCTTTGTTTGATGCAACTGGCCGACGTTTTCGGC
AGGTGCCTTTTACACCGGATGTGGTCCGGGCCGCGCTGGCTGCTACGCCCCAAATG
GCGTAG
>*alfp_2915*
ATGAACCCTTGTACTCGCCCGCATACTTTGCGGGTCAATCAAGAGACCCACATCATC
GAGGTGGAGCCCGATACGCCGCTGCTTTATGTATTGCGTAACGACCTGGAACTGAAT
GGCCCCAAGTTTGGCTGCGGCCTGGGTGAATGTGGCGCCTGTACGGTGCTGGTCGA
TGGTGTGGCCGCCCGCTCCTGCGTCGTGCCGGTCAGCCTGGTAGAAAACCGCCAGA
TCACTACGTTAGAAGGCCTGGCCCGAAATGGCCAGCCTAATGATGTTCAGCAGGCAT
TTATAGATTGCCAGGCTGCGCAATGCGGCTACTGCCTCAATGGCATGGTCATGACCG
TGCAGGCCCTGCTGGAACGCAATCCGCAAGCCACCGAAGAGCAAATTCGGGACGA
ACTGCGCTATAACCTGTGCCGTTGCGGCACGCACGTAGAAATCATGCAAGCCGCCGT
ACGCGTCGCAAAGGCTCGGCAATGA
>*alfp_2916*
ATGAGTCATTCAGTACAAGACCTTTCCCGCGCGCTGTGGCCGGAACTGCCTGCGGG

CACCAGCTTGCTGCATGGCGCTGTGGTGCGCCCCCCTTACTGGTCCTATGAAAACGG
GCAGTATCTGGGCGCTGAATTACAAATGGTAAACCAACAAGCTGCCCTGCAAGTAC
CCGGCGTTGTAGCCTGTGTGCATATGGGCAATTTCCTGGGTGTGCTGGCGGTACAAG
CCGAGCAGGCTCAACAAGGCGCCGCCTTGCTGGATGCGCGCTGGGCCACGCCTGTC
GCTGCAAACCAGGCTGCCCTGCCAGCCGATGAGACTGTTGCCGCGACACTGGGCTC
CCCCGATCAAAGCTATGAGTGGCACTCTGCCGCCGAGCCTCAGGAAACCGCTTGGG
CCCGTGCTTGTTATCACGACAAGCAACTTTATGTCTGGGCGCATACGCAGCGCCCCG
CTGCCCTGCTGATTGAGCTGCAAGCCTTGAGCGGCCTGCCCAGCGAACAGATCCAT
TTGCAGGATATTGCCGGCAATCAGGCCGATGCCTACGACTGCGCCATGGATGCAGCC
GTCATGGCCTTTGGCCGCCCCCAGGCCGTGCAAGTTCGCGCCAGCCACAGAGAAGC
CACCATTCGCCTGAGCGTCTATAAAGGCACCGCAGAGCGAAACGACTTGAATGCCC
ATTTGCGGGCGACCCGCTGGCAACTGAACACCTTGTCCGGTGCACGTCCTTCGCTG
GCAGCCATTCTTTGCGGCCAGCCAGGTTTGCCCAGCAGCGGTCCGGACGTAAAGAG
CGATTACTTTTCTGCACCCACGCCTAACTATGATGGCGCGGCCGCCAGCAGTGACCC
CGACAGTCTGGCCCAGGCCACAGTCTTTGCCCAGGAGTCGCAGTTCGATCAGGACT
GCCACAGCCTGGGTCTGGACCCTTTGGAGGCACGACTGGAACAAGTCAGCAGCCC
CCAAGGTCGAGAACTGCTGCAACGGGTTGCCGAGCAATCGGACTGGTCCGAGCCCT
TGCCCGCCCATCAAGGCCCTTTGCGCAAGGGTCGCGGGCTGGCCTACAGCCATATTG
TCGAGAACGTCCCCGGACAGGCTGCACGAGAACAGTGGTCCGCCTGGGCCGTGGA
TGTCAGCGTCGATACCCGTCAAGGCACGCTCAGCATAGACAAGCTGACCATTGGCC
ACGACAGCACCGAACTGAGCAATCCCGAGCAGCCCCCGGAGTCTGCGCCCGCCCT
GGCCGACCGACTGGGCCGTTGGGCTCAACAACTGCTGAACAATGGCGTGGGCAAA
GGCGGCGAAGGTAGCAGCGACAGCCCTGTCCAAGAAACCGCCGACAAGCCCGCCG

TCCAGTTGGTCAAACGAGAGTCTGCCGTAGGCCAGCCTTTGGCCTGGAACCAGGGC
GTGGAACTGCCTGCCGCGGCGGCCATTGCCAATGCAATTTTCAATGCCAGTGGCATA
CGCCTGACCAGTGCGCCTTTTAGTGAGCAATCTCTGGCGCTGGGCTATCAGTCCGAT
AAAACAGGCAGCAGCAAAAAACGCAAAGCCTGGTGGGGAGCCTTGGCCGCGGTCG
CCACCGGCACAGTCTTGAGTGCCCTGCCCTGGCGGCCCGCCATCGCGCCCGTCGGC
CAAGTGGACACATCCATCTTTTCAGAGCTGGCGATTGAGCGTGGCCGCCTGGTTGCC
ATTGCAGGCGATTGCATGGTCTGCCACACCGCTGAGGGCGGCACACCCAATGCGGG
CGGTCTGGGCCTGGATACACCGTTTGGCACTATCTACACCACCAACATCACACCGGA
CAAAGAAACCGGCATTGGTAGCTGGAGCTACAAAGCCTTTGAGCGCGCCATGCGCG
AGGGCATCCATCAGGACGGACGCCACCTGTACCCGGCCTTCCCTTACACGGCCTTTG
CCAAGATCAGCGATGAGGACATGCAGTCCCTGTACGCCTACTTGATGACACAAGAG
CCAGTGAAGTCCGAAGTGCCTGAAACCAAGCTGCCTTTCCCCATGAATATGCGGCC
CCTGGTGGCGGGCTGGAACCTGCTGTTTCACCGTGATCCGAATGCCTACGTGCCGGA
TCCGACGCAAACCGTGCAATGGAACCGGGGGGCCTATCTGGTCAATAGCAGCGGTC
ACTGTGCGGCGTGCCATAGCCCACGCAATATGCTGGGCGCGGAAAAAGGCGGTAAA
GCGAATTTCCTGGCTGGTGGTTTTGCGGACAACTGGGAAGCGCCGGCGCTCAATAG
CCTGTCCAAAGCCCCCATCCCCTGGACAGAACAGGAGCTGTACCAGTACCTGCGTA
CCGGCTACTCGCCCCGTCATGGCGTGGCGGCGGGACCGATGGGGCCGGTCGTCGCA
GGCTTGGCAGAACTGCCGGAGTCCGACGTCCGTGCCATGGCGCACTACCTAAGCAG
TTTGAACCCGGTCGAGAGTGAACAAGAACAAACACACGCCGCCCAGGCCGCGCTG
CTGGAGCAAGATAGCCGCAGCAATAAGGACGTGATGGTGATGCCAGGCGAAAACCT
GTTCAACGGCGCTTGTGCTGTTTGCCATGACCCACGTGGTGGGCCGGTGCTCTTTGG
GGCTCGCCCGTCCCTGGCCTTGAACAGCAATCTGCACAGTGAACACCCGGACAATA

CGATTCAGGTGTTGATGCACGGCATTACCCGTCCGGCACAGCCCACCCTGGGTTCCA
TGCCCGGTTTCAAGAACAACATGAATGACGAGCAGATGGAAGATCTGCTGAACTATA
TGCGGGCGCGTTTTGCACCGGACAAACCCGCCTGGACAGGCCTGAAGGACAAGAT
TGCGACCATACGGGAGCAGAAGGGGCATCTGTAG

>*alfp_3272*

ATGAAAACCTACCGCATCGGCCAGATCGTTCCCAGCTCCAACACCACTATGGAAAC
CGAGATTCCTGCCATGTTGCAGGCTCGTTACGCCGAGTTTCCTGAAGAACGCTTCAC
CTTCCACTCCTCGCGCATGCGCATGATGCACGTGAACCCCGAAGAGCTGAAAGCCA
TGGACATTGCCAGCGACCGCTGCGCGGTTGAACTGAGCGACGCCCGTATGAGCGTG
ATGGCCTACGCTTGCCTGGTTGCCATCATGGCCCAAGGCGACGGCTACCACCGCGTC
TCGCAAGCCCGTCTGCAAAACACCGTTAAAGAAAACGGCGTGGAAATTCCTGTGCT
GAGCTCGGCCGGTGCCCTGGTCGATACGCTGAAAGAATTCGGCTACAAGAAAGTCT
CCATCATCACCCCTTACATGAAACCGCTGACCAAGCGCGTGGCTGACTACATCGAAG
CTGAAGGCATCGAAGTTCAAGACTCCATCAGCCTGGAAGTGTCGGACAACCTGGAA
GTGGGCCTGCTGAACCCGGAAAACCTGCTGGAGCACGTCAAACGCCTGAACCACG
ACGGCGTGGACGCGGTCATCCTGTCCGCTTGCGTACAGATGCCTTCCCTGCCCGCCA
TCCAGCGCGCTCAAGACCAGATCGGCAAGCCCGTTCTGTCCGCTGCTGTTTGCACC
GTGTACCAAATGCTCAAGACCCTGGGTCTGGAAACCCGCGTACCTAACGCTGGCCA
CATCCTGTCGGGCGCCAAGCCACAAGCCTGA

>*alfp_3273*

GTGCAGATCAGCAAAAAAAAGCGATATGATGCCTGCATGAAACTGGACCTGCTGAC
CCTCAAACTGTTTGTCCGCATACTGGAAGAAGGCACGATCACCCAAGCGGCGGAGC
GTGAGCATATTGCTGCGGCTGCGGTCAGCCGCCGGATTGCAGATCTGGAACAGTCTC

TGAACACCACCTTGCTGCTGCGTACGAACAAAGGTGTCAGCCCGACCGCAGCAGG
CCTGGAACTGCTGTACCGCTCGCGCGCATTGCTCAATAGCGCGCAAGAGATCGAAA
CGCGCCTCCAGGCGTTCTCGCAAGGCCAGCAAGGCCTGGTGCATATCCTGGCCAAT
ACATCGGCTATTTCTCAGTTTCTGGCCGAGCCACTGGGCGAGTTTGGACGTCTGCAC
CCGGCTATTCCCCTGCAACTGGAAGAACAAACCAGCCTGGACATTATTCGGGCCCT
GGCAGAGGGTAAAGCCGATCTGGGAGTATTTACCCGCCTGCCCTACGCGGCCGACA
TCGAGGCCTATCCTTTTCGCAGTGACAAGCTGGTCGTGCTGGTGCCTCTCAATCACC
CCTTGGCTCAGCATGAAAAAATCCGCTTCGAGCAAACGCTGGAGCATGAGCAGATC
ACGCTGCTAACGGGCACGCAGCTGCATTACCAGATCACCAAAATCGCCATGGAAGC
CAACCGCTCCGTGCGCATTCGCACCGAAGTGTCGGGCTACGACGCCATGTGTTTGCT
GATTAATGCAGGCATGGGTATTGGCATCTTGCCGCGCAAGAGCGCCAGTATTTATCA
GATCCCCAATACCCGTGTGATTGAGCTGGACGAAGAGTGGAGTCAGCGAGAAATTC
TGATCGGTGTGCGCCGCCGCAGTGACCTGCAACCGAGCGCAGAAAGCTTGCTGAGC
TTCCTGCTCGAGAGCGGCGCTTAA

Full-length blots of TLC

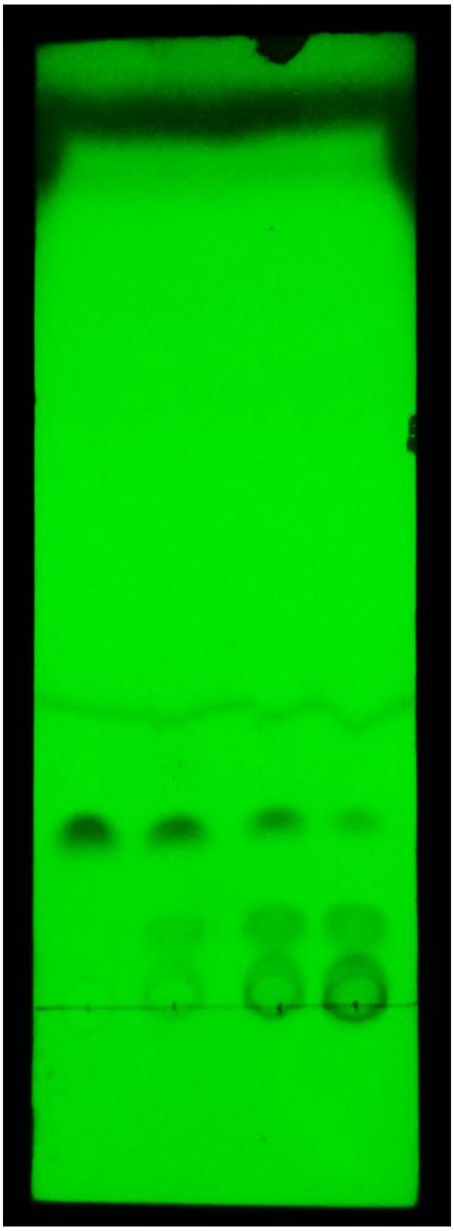

Supplement: Supplementary file 1 — Suppnementary information [file 41598_2019_40199_MOESM1_ESM.pdf]
